# Enhanced durability of round bamboo treated with copper naphthenate under heat-cold impregnation

**DOI:** 10.1098/rsos.220247

**Published:** 2022-11-23

**Authors:** Jun Jiang, Shuaibo Han, Xin Ren, Hui Wang, Hongwei Yu, Fangli Sun

**Affiliations:** ^1^ College of Chemistry and Materials Engineering, Zhejiang A&F University, Hangzhou 311300, People's Republic of China; ^2^ Huzhou Inspection and Testing Center, Huzhou 313009, People's Republic of China

**Keywords:** bamboo, preservative treatment, decay resistance, mould resistance

## Abstract

Round bamboo has aroused much interest in construction for its mechanical properties, but poor biological durability seriously restricts its application. In order to develop a suitable and effective preservative treatment method for round bamboo, copper naphthenate (CuN) was adopted and impregnated into round bamboo using heat-cold procedure. The distribution and retention of copper naphthenate in round bamboo were studied, and the biological durability represented by the mould and decay resistance were investigated. The results showed that the retention and fixation of copper reached 0.39 kg⋅m^−3^ and 85.3%, respectively. Scanning electron microscopy–energy dispersive X-ray spectrometry further disclosed an increasing trend in the composition of CuN from the end inward. X-ray photoelectron spectroscopy and Fourier transform infrared spectroscopy analyses later revealed that CuN could be fixed on bamboo in the form of hydrogen bond or complex reaction. Statistical analysis showed that the increasing concentration of CuN from 0.3% to 0.5% and 0.8% (calculated as Cu^2+^ content) has significant contribution against *Trametes versicolor* and *Gloeophyllum trabeum* in comparison with the untreated bamboo. Meanwhile, when the concentration of treating solution increased to 0.8 wt%, the resisting efficacy for *Aspergillus niger*, *Penicillium citrinum* and *Trichoderma viride* soared as high as 85.9%, 94.8% and 70.3%, respectively.

## 1. Introduction

Bamboo as fast growing woody grass has aroused increasing attention for its sustainability and extensive applications in daily necessities and constructions. However, round bamboo has numerous unique advantages which have not been well developed. It is rich in culture connotations, special in the cylindrical and hollow shape, possessing smooth, hard and hydrophobic epidermis. Round bamboo has attracted great interest in building. As a building material, it meets the requirements for mechanical strength very well, but frequently suffers from mould and decay, as well as splitting and cracking, which causes great losses in the original appearance and strength [1].

To protect bamboo from detrimental fungi, various methods were used, mainly including water leaching, painting, smoking, heat treatment and preservative treatment [2]. Unfortunately, the first two methods only provide limited durability and are usually not durable for outdoor application. Smoking can be applied to protect round bamboo from fungi and insects, but it is a time-consuming procedure which generally takes several months and the colour of bamboo changes to bronze or dark yellow, which is not accepted in some applications [3]. The traditionally high-temperature treatment for bamboo strips and slices in a saturated steam atmosphere is unsuitable for round bamboo due to the problem of cracking [4].

Impregnation of round bamboo with preservative solutions such as alkaline copper quaternary (ACQ), copper azole (CuAz) and organic biocides formulations are commonly used at present, but have low chemical penetration and distribution abilities [5]. The combination of polyethylene glycol (PEG)/boron and wax heat treatment has been used to improve the cracking and decay resistance of round bamboo [2,3], while the leaching of both PEG and boron are still not solved. To meet the requirement for diverse applications, it is necessary to find a feasible way to introduce the fungicide into the inner part of round bamboo and simultaneously enhance its leaching resistance.

Vacuum-pressure impregnation is a common and efficient method for wood preservation [6–8], in which wood is dried to a moisture content (MC) around 20% before treatment. However, the harvested round bamboo with the MC over 50% greatly reduced the penetration of preservatives. Additionally, the closed internode brings the risks of cracking during pressurization [9].

Oil heat treatment is a green and effective method for bamboo modification. It is reported that round bamboo heated in tung oil could improve both the dimensional stability and mould resistance [10]. The combination of heat treatment with preservatives absorption would improve the properties of round bamboo significantly. Heat-cold treatment is suitable for round bamboo because not only can it dry the round bamboo to MC 20–30%, the required moisture content for preservative treatment, but also create a temporary vacuum in the round bamboo which will draw the preservatives into the inner part, therefore, giving the bamboo prolonged resisting effect against fungi [11,12]. Copper naphthenate (CuN) is an oil-borne preservative used from the beginning of twentieth century [13], which penetrates wood well and provides wood with both stability and decay resistances [14]. However, few reports addressed the introduction of CuN into round bamboo under heat-cold treatment.

The objective of this research was to investigate the effect of heat-cold impregnation in round bamboo treatment, as well as the decay and mould resistance of CuN in bamboo preservation. Based on the research, an effective and suitable method of practical use for round bamboo was obtained.

## 2. Material and methods

### 2.1. Materials

Four-year-old bamboo (*Phyllostachys iridescens*), growed in yellow-red loam soil, sourced from Anji County (latitude 30°38‘17.66″ N and longitude 119°40′55.88″ E), at an altitude of 200 to 500 m, Huzhou City, Zhejiang Province of China, was cut into round bamboo segments of 2 m long from 2 m above the base of the bamboo, the initial average moisture content was 71.9%. Subsequently, three dimensions of specimens, free of nodes, mildew and insect holes, were prepared according to the test items and conditioned at room temperature (approx. 22°C) with relative humidity of approximately 65% for a week. To determine the content and distribution of copper penetrated in round bamboo culm, specimens with dimensions of 50 ± 4 mm (diameter) × 200 mm (length) were prepared. The leaching and decay resistance tests were conducted on blocks cut from the bamboo culm with dimensions of 20 mm (length) × 20 mm (width) × 4.6 mm (thickness, average), and mould resistance test with dimensions of 50 mm (length) × 20 mm (width) × 4.6 mm (thickness, average). The specimens maintaining the moisture content of 60.3% were used in the succeeding treatments and six replicates were prepared for each treatment.

Commercial silicon oil (colourless transparent liquid) for bamboo heating and food-grade mineral oil (mineral oil) as solvent for CuN were purchased from the local company in Hangzhou, Zhejiang Province, China. Copper naphthenate (8% Cu^2+^ content) sourced from Strem Chemicals, Inc. was used without purification. The preservatives in this study were prepared by dissolving copper naphthenate into mineral oil, with concentrations of 0.3%, 0.5% and 0.8% (calculated as Cu^2+^ content).

*Aspergillus niger* (*A. niger*, isolation number: GDMCC 3.411), *Penicillium citrinum* (*P. citrinum*, isolation number: GDMCC 3.458) and *Trichoderma viride* (*T. viride*, isolation number: GDMCC 3.140) were considered as the main reasons for the bamboo mildew and selected as test fungi. *Trametes versicolor* (*T. versicolor*, isolation number: GDMCC 3.383) and *Gloeophyllum trabeum* (*G. trabeum*, isolation number: GDMCC 5.248) were used as decay fungi. These test fungi were provided by Guangdong Institute of Microbiology (Guangdong Province, China) and archived by the National Engineering Technology Research Center for Comprehensive Utilization of Wood Resources (Zhejiang Province, China).

### 2.2. Preservative treatment

**Figure 1.**
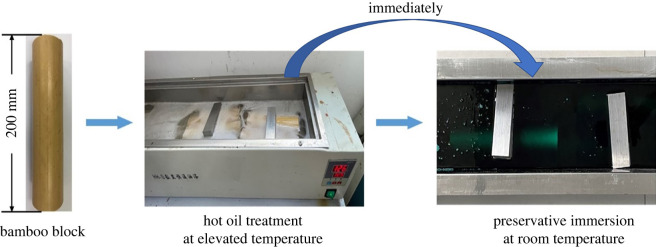
Treatment of bamboo under heat-cold procedure.

Bamboo culm (200 mm in length) with the average moisture content 60.3% were treated using heat-cold procedure (figure 1). Firstly, the specimens were immersed in silicon oil at an elevated temperature from 60°C to 80°C, then 100°C and finally 125°C at 30 min intervals. Subsequently, the specimens were transferred immediately to the preservatives and impregnated for 30 min at room temperature 25 ± 2°C. The treated specimens, after being conditioned for 24 h, were then dried at 60 ± 3°C for 12 h, and ready for the leaching, decay and mould resistance tests. The hot oil alone treated controls (HSO) were the specimens free from the second step impregnation, and the combination of heat-cold treated controls (HTO) were those heated in silicon oil then immersed with mineral oil alone. Blocks numbered 0.3 CuN, 0.5 CuN and 0.8 CuN were those submerged into CuN solutions at the concentrations of 0.3%, 0.5% and 0.8% immediately after the hot oil treatment. The untreated bamboo blocks were termed as the controls.

To understand more about the retention and distribution of copper naphthenate in bamboo blocks, inductive coupled plasma emission spectrometer (ICP) was applied to determine the content of copper ion. The treated specimens were oven dried at 60 ± 3°C for 12 h, then cut into pieces and milled to 60 mesh. Approximately 0.1 g bamboo powder was digested in the mixture of 4 ml concentrated nitric acid (68%) and 1 ml hydrochloric acid (37%) at an increasing temperature from 80°C, 150°C to 200°C at 10 min intervals until dissolved and transparent. The content of copper was determined by ICP (*C_0_*). The retention of CuN (calculated as copper*, R*, kg m^−3^) in each specimen was calculated according to equation (2.1), and the average retention was evaluated at last.

2.1 $$\mathrm{Retention}=\frac{C_{0}}{V},$$

where *C*_0_ is the amount of CuN (calculated as copper) in the specimens (mg), and *V* is the volume of specimens (mm^3^).

To investigate the fixation of CuN in bamboo, the treated blocks were subjected to a water leaching procedure according to American Wood Protection Association (AWPA) E11 [15]. The leaching test was conducted on bamboo blocks with dimensions of 20 mm (length) × 20 mm (width) × 4.6 mm (thickness, average) in deionized water at room temperature for 14 days. Six pieces of bamboo blocks with the same treatment were firstly submerged in water under vacuum (−0.08 MPa, 20 min). After 6, 24, 48 h and thereafter at 48 h intervals, the leachate was removed and replaced with 300 ml fresh water. At the end of leaching, the bamboo blocks were air dried for 24 h followed by vacuum dried at 40°C for 3 days. The amount of copper in the blocks before (*C*_0_) and after leaching (*C*_1_) was determined by ICP. The fixation of CuN (calculated as copper, *F*%) was obtained from equation (2.2).

2.2 $$\mathrm{Fixation}\;(\text{\%})=\frac{C_{1}}{C_{0}}\times 100\text{\%}.$$

### 2.3. Scanning electron microscopy–energy dispersive X-ray spectrometry analysis

The morphological properties of both the untreated control and the highest concentration of CuN 0.8% treated bamboo (0.8 CuN) were recorded on a SU8010 scanning electron microscope (SEM) at an accelerating voltage of 3 kV. The transverse, tangential and radial sections of the samples were prepared by microtome to get well-prepared bamboo surfaces for electron microscopy.

**Figure 2.**
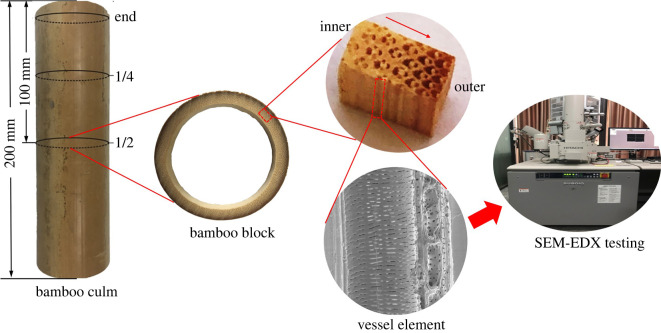
Scheme of sample preparation and SEM-EDX analysis.

In order to obtain information about copper distribution along the treated bamboo, subsamples were sliced from the centre of bamboo culm located at the end, quarter and middle of the longitudinal direction (figure 2). The elemental composition of bamboo samples was analysed by the electron dispersive X-ray spectroscopy (EDX).

### 2.4. X-ray photoelectron spectroscopy and Fourier transform infrared spectroscopy analysis

The untreated controls (control), the hot oil alone treatments (HSO), the heat-cold absorption of mineral oil (HTO) and 0.8 CuN treatments were collected and milled to fine powder for X-ray photoelectron spectroscopy (XPS) and Fourier transform infrared spectroscopy (FTIR) analysis. The XPS experiment was conducted on Thermo Scientific K-Alpha XPS equipped with a monochromated Al K*α* X-ray source (1486.6 eV), at a power of 12 kV and 6 mA.

FTIR spectra were recorded on a Thermo Scientific Nicolet 10 type instrument. The treated and untreated bamboo were crushed and dispersed in potassium bromide (KBr) at the ratio of 1 : 100, followed by compression under 16 MPa to obtain thin pellets. Spectra were recorded in the range of 4000–400 cm^−1^ at a resolution of 4 cm^−1^ and 32 scans.

### 2.5. Decay and mould resistance tests

The decay test was carried out referring to AWPA E10–16 [16]. The untreated controls (control), the hot oil alone treatments (HSO), the heat-cold absorption of mineral oil (HTO) and three different concentrations of CuN treatments were oven dried at 60°C for 2 h, 80°C for another 2 h and then 105°C for 8 h. The decay test was carried out in the culture bottles with soil blocks substrates. Approximately 150 g soil was added in 250 ml wide-mouth screw cap bottles with 26 ml water to each culture bottle. By calculation, the soil substrate with a water-holding capacity was 30.9%, and pH was 6.2. Before inoculation, the test fungi including *T. versicolor* and *G. trabeum* were purified and activated. After the feeder strips were covered with decay fungi, the test blocks were placed on the top of them. The culture bottles containing the test blocks were moved to the incubation cabinet set at 28 ± 2°C and the average relative humidity (RH) 78 ± 5%. After 12 weeks incubation, the blocks were taken out, brushing off the mycelium carefully, then air dried for 48 h. The same oven drying procedure was conducted at 60°C, 80°C and 105°C, respectively. The mass loss (ML, %) was calculated from the weight before (*W*_1_, g) and after (*W*_2_, g) decay test (equation 2.3).

2.3 $$\mathrm{Mass}\;\mathrm{loss}\;(\text{\%})=\frac{W_{1}-W_{2}}{W_{1}}\times 100\text{\%}.$$

Mould resistance of test blocks with dimensions of 50 mm (length) × 20 mm (width) × 4.6 mm (thickness, average) were performed referring to AWPA E24-21 [17]. *Aspergillus niger*, *P. citrinum* and *T. viride* were selected as the mould strains. The experiment was conducted in Petri dishes with potato dextrose agar (PDA) substrate. After inoculating the spore suspension on PDA substrates, U-shape glass bars were placed gently on top of the fungi-bearing substrate and the specimens were then carefully put on the bars. The Petri dishes containing test blocks were moved to incubation cabinet setting at 25 ± 2°C and 85 ± 5% RH. Mould resistance effects of six replicates were evaluated visually and scored using a scale of 0 to 5 grades. Resisting efficacy (RE) of bamboo against test fungi was computed as equation (2.4).

2.4 $$\mathrm{RE}\;(\text{\%})=\left(1-\frac{P_{1}}{P_{0}}\right)\times 100\text{\%},$$

where *P*_0_ and *P*_1_ are the average scale of fungal infection on the untreated controls and the treated blocks, correspondingly.

The data of decay test were subjected to statistical analysis. Statistical differences (*p* < 0.05) among the different treatment groups were evaluated using one-way ANOVA followed by uncorrected Fisher's LSD multiple comparisons test. GraphPad Prism v. 8 (GraphPad Software, San Diego, CA) was used to perform data analysis.

## 3. Results and discussion

### 3.1. Impregnation and distribution of copper naphthenate in round bamboo

CuN preservative containing a minimum of 1.0% copper metal are recommended for wood product, such as poles, piles and lumber in AWPA M4 standard [18]. Considering the better decay resistance of bamboo in comparison with wood [19,20], and ensuring proper viscosity of CuN to achieve good penetration, as well as balancing the cost and efficiency of treatment, lower than recommended amount of CuN for wood was applied in this experiment (table 1). The retentions of copper in bamboo samples were positively dependent on the concentration of treating formulation. Although the uptake of CuN in round bamboo increased with the increment of concentration, the retention was still as low as 0.09–0.39 kg⋅m^−3^ (calculated as copper content), which is much lower in comparison with soft maple 2.24–9.77 kg⋅m^−3^ and red oak 2.14–3.84 kg⋅m^−3^ at the same concentration [13]. The low retention was ascribed to the unique structure of round bamboo and the heat-cold impregnation method other than vacuum-pressure treatment. As for the structure of bamboo, it lacks transverse conducting rays, and the dense and smooth epidermis are adverse to preservative penetration.

**Table 1.** Retention and fixation of CuN in the treated bamboo.

concentration of CuN^a^ (%)	retention^a^ (kg⋅m^−3^)	fixation after leaching^a^ (%)
0.3	0.09 (0.006)	65.5
0.5	0.20 (0.009)	77.9
0.8	0.39 (0.009)	85.3

^a^Note: calculated as copper content; the values in the parentheses are standard deviations.

As far as the commonly accepted vacuum-pressure treatment in wood or bamboo strips, it is unsuitable for round bamboo treatment due to a series of practical and technical problems. Firstly, round bamboo for construction is normally in various lengths, from 7–8 m to as long as 30 m and is separated by nodes, where the cells are arranged differently from the internodes. Although there are suggestions on punching the nodes to enhance the penetration of modifiers, the strength will be reduced and the cracking will be accelerated [11,21]. Secondly, they are generally high in moisture content due to the lack of high quality and efficient drying technology. The newly cut round bamboo, whether or not it has been pretreated with NaOH solution, has difficulty in reducing the MC below 20% in a feasible time span with high quality. The commonly used air drying is not only time-consuming, generally 6 to 12 weeks, but also has high risks of cracking and splitting, while kiln drying has not been widely used in round bamboo [22]. The high moisture content in bamboo adversely influences the effect of vacuum-pressure treatment. Lastly, the waterproof properties of the inner (pith ring) and outer layers (epidermis), and the separate and closed bamboo internodes increased the risks of cracking and splitting during the vacuum-pressure treatment. Therefore, although heat-cold treatment is not so effective as vacuum-pressure treatment in preservatives impregnation, it is more practical at present.

Despite the insoluble property of CuN in water, a 14-day leaching experiment was conducted to predict the performance of CuN in protecting bamboo in rainy seasons. Table 1 presents that the fixation of CuN increased with the increment of retention, and the top fixation ratio 85.3% was obtained at the retention of 0.39 kg⋅m^−3^. This proportional increase of fixation to retention was also found in other copper-based preservatives [23]. A number of literatures have proved that copper can be bound to wood, including CuN [24]. In our view, the water-insoluble CuN is resistant to water leaching, and this perception was proved by Hein [25,26], who used tap water to leach fuel oil-based CuN for 30 days and rinse CuN-filled soil column for one year, while little Cu was detected.

But in our experiment, more than 14.7% CuN was leached from bamboo, which is out of our expectation. It is reported that the leaching resistance of CuN was affected by both the carrier and leaching medium [27], and accordingly, the leaching could be partially ascribed to the carrier in the CuN formulation. In addition, the unique structure of bamboo is also responsible for the leaching of CuN. In the absence of transverse cells, bamboo is mainly composed of longitudinal vessels (metaxylem and protoxylem), fibre, parenchyma cells and phloem, which facilitates the longitudinal penetration. It is accepted that the vessel of *Phyllostachys Iridescens* averaged to 834.6 ± 202.0 µm in length and 125.9 ± 19.7 µm in diameter [28]. The rich and fluent longitudinal tissues of bamboo probably advanced both the access and leach of preservatives.

**Figure 3.**
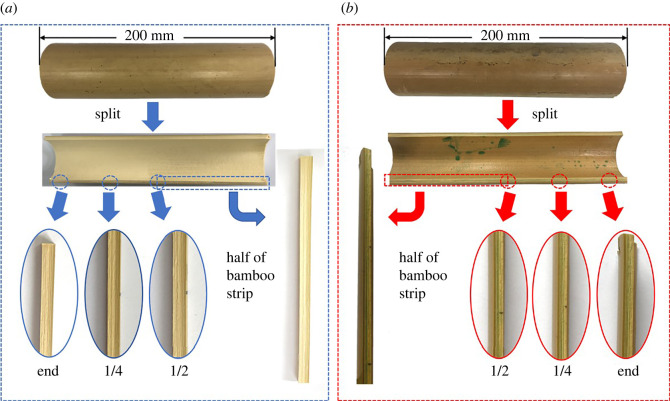
Comparison between the distribution of CuN in the three positions (1/2, 1/4 and end) of control sample (*a*) and treated bamboo (*b*).

When splitting the round bamboo and checking the distribution of CuN, it was found that CuN stuffed the cavities along the axial direction (figure 3). The middle of the bamboo culm even filled with more CuN than that of the quarter of the culm, which was beyond our expectation but suggesting a high efficiency of longitudinal permeability.

The SEM-EDX was used to investigate in detail on the distribution of CuN in bamboo, as well as the amount of silicon oil retained in bamboo during the heating procedure.

Results in table 2 confirmed the upward trend of Cu deposition from the end of bamboo culm to 1/4 length and inward to the middle. The higher amount of CuN in the inner part of bamboo culm could be attributed to the heat-cold treating procedure. Round bamboo, when oil heated from 60°C to 125°C, the air and water in it were driven to dissipate along its unique longitudinal channel. The continuous migration of air and water not only make room for preservatives but also clear the pathway for the subsequent penetration. When the heated round bamboo was immersed immediately in cold CuN preservative, the air and water in it were suddenly condensed and a temporary vacuum would be produced, which promoted the penetration of preservatives. As the temporary vacuum in bamboo was formed and maintained under 125°C, the cold suck procedure should be well arranged to ensure a prompt and timely introduction of CuN preservative.

**Table 2.** Elemental analysis on CuN treated bamboo by SEM-EDX.

location	copper (%)	silicon (%)
end of bamboo culm	0.18 (0.03)	3.39 (0.77)
a quarter of the culm	0.33 (0.11)	1.35 (0.42)
middle of the culm	0.81 (0.28)	2.01 (0.06)

Note: the values in the parentheses are standard deviations.

The reduced content of CuN from the middle outward till the end of bamboo culm in table 2 was probably caused by the drop of surface temperature and partially released vacuum during the cold immersion procedure.

A higher distribution of silicon was detected at the end of bamboo culm, which is understandable because silicon oil is large in molecular weight and difficult to go inside. But to our surprise, it could not only penetrate into the inner part but also presented an increased amount from the 1/4 length to the middle. This phenomenon testified that silicon oil could penetrate bamboo during the oil-bath procedure, which agreed with the result of tung oil [29].

**Figure 4.**
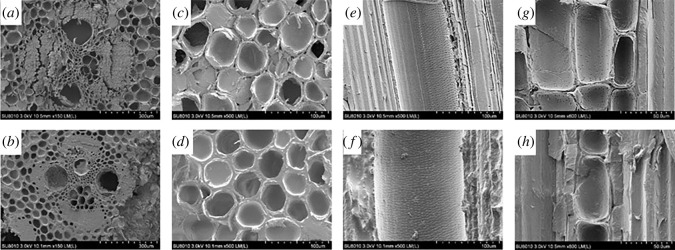
SEM morphology of untreated control (*a,c,e,g*) and 0.8 CuN-treated bamboo (*b,d,f,h*).

To reveal the microscopic distribution of CuN in bamboo culm, the morphology of treated bamboo was observed under SEM and the results are shown in figure 4. The transverse section of 0.8% CuN treatment (0.8 CuN, figure 4*b* and *d*) presented a dense and ordered structure in comparison with the untreated control (figure 4*a* and *c*). The intercellular space between parenchyma cells of 0.8 CuN disappeared and some vessel pores were filled with oil substance. The microstructure of vessels on the radial section (figure 4*e* and *f*) and parenchyma on the tangential section (figure 4*g* and *h*) presented the state of pits. The untreated bamboo (figure 4*e* and *g*) displayed clear and empty pits, while the 0.8CuN (figure 4*f* and *h*) treated blocks showed obscure and partially filled pits, suggesting that oil and preservatives might penetrate into the inner part of bamboo from the vessels and diffused into the adjacent parenchyma cells.

### 3.2. X-ray photoelectron spectroscopy analysis

**Figure 5.**
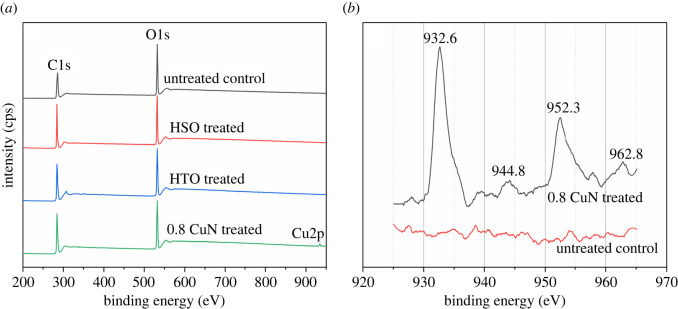
XPS spectra of bamboo. (*a*) XPS profile of untreated control and treated bamboo; (*b*) Cu2p spectra of untreated control and 0.8 CuN-treated bamboo.

XPS measurements were performed on the untreated control, the hot oil alone-treated controls (HSO), the heat-cold absorption of mineral oil (HTO) and the blocks treated with 0.8% copper naphthenate, respectively, to provide information on the oxidation and chemical bonding state of elements. The results in table 3 and figure 5 show that copper was detected in blocks of 0.8 CuN, mainly as the Cu^2+^ state with the binding energy (BE) of 932–963 eV (figure 5*b*), indicating that CuN could penetrate into round bamboo under the heat-cold impregnation [30].

**Table 3.** Results of XPS analyses.

sample	C_1s_ (%)	C (%)				O_1s_ (%)	Cu_2p_ (%)	O/C
		C1	C2	C3	C4			
untreated	64.2	34.2	51.6	12.4	1.9	35.7	–	0.56
HSO	66.5	82.5	14.1	3.4	–	33.4	–	0.50
HTO	67.3	67.8	24.6	7.6	–	32.7	–	0.49
0.8 CuN	67.8	66.6	26.5	7.0	–	31.8	0.4	0.47

Note: untreated: the untreated control; HSO: the hot oil alone treated blocks; HTO: blocks treated using the heat-cold absorption of mineral oil; 0.8 CuN: blocks treated using the heat-cold absorption of 0.8% copper naphthenate.

The ratio of oxygen to carbon (*O/C*) decreased slightly for the treated blocks in comparison with the untreated control, and in the order of HSO, HTO and 0.8 CuN. As the blocks were treated in silicon oil and at a relatively low temperature (less than 125°C), the trend for oxidization might not happen. High resolution scans of the C1s region were analysed after curve fitting and the results are shown in table 3. The amount of C1 characterizing C–C or C-H increased greatly in the treated blocks, while C2 (C–OH), C3 (O–C–O or C=O) and C4 (O–C=O) declined dramatically, especially for CuN samples, where the C4 bond even disappeared. As CuN contains COOH and COO^−^ groups, the absence of C4 suggested the reaction of CuN and bamboo. According to equation (3.1) developed by Matuana and Kamdem [31] for the computation of the ratio of oxidized-to-unoxidized carbon atoms, the oxidation had not occurred on the treated bamboo (the ratio of oxidized to unoxidized below 1%), which agreed with the results of *O/C* ratio.

3.1 $$\frac{C_{\mathrm{oxidized}}}{C_{\mathrm{unoxidized}}}=\frac{C2+C3+C4}{C1}.$$

### 3.3. Fourier transform infrared analysis

**Figure 6.**
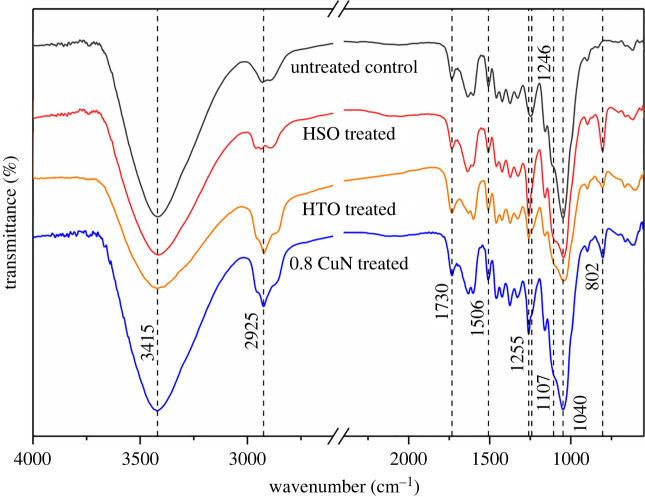
FTIR spectra of untreated and treated bamboo.

To characterize the chemical changes of bamboo as well as the interaction between CuN and bamboo under different treatment, the chemical structures of the untreated control, HSO, HTO and 0.8 CuN were analysed by the FTIR spectroscopy and the corresponding spectra are shown in figure 6.

The bands at 3415 cm^−1^ correspond to hydroxyl groups present in the bamboo. Both HSO and HTO displayed a blunt and decreased peak of -OH in comparison with the untreated control, indicating that the two treatments enhanced the formation of hydrogen bond between hydroxyl groups [32]. The band at 2925 cm^−1^ assigned to CH_2_ stretching was more pronounced in HTO and 0.8 CuN, which was contributed by the mineral oil and CuN containing alkanes and cycloalkanes. The peaks at 1730cm^−1^ assigned to C=O were not prominent for sample 0.8 CuN comparing with the control and the other two treatments, and the signal at 1506 cm^−1^ characterizing lignin slightly shifted toward higher wave number, which might be attributed to the combination of copper naphthenate and lignin [33–35]. The C–O stretching vibration of ether bonds appearing at 1255–1246 and 1040–1107 cm^−1^ changed significantly, which suggested the interaction of CuN and bamboo components. These observations can be related to the ligand exchange between CuN and bamboo components, which contain rich carbonyl (C=O), carboxyl (COOH) and hydroxyl (OH) groups. A new peak at 802 cm^−1^ assigned to Si-C of silicon oil appeared on HSO, HTO and 0.8 CuN sourced from the heating oil [33].

### 3.4. Decay resistance

The untreated controls were severely attacked by both white-rot fungus (*T. versicolor*) and brown-rot fungus (*G. trabeum*) after 12 weeks decay test, with the average mass loss (ML) as high as 19.5% and 21.4%, respectively. It is also found by other researchers that bamboo is less resistance to brown rot fungi than white rot fungi, which could be ascribed to the chemicals the two fungi degrading [14,36,37].

**Figure 7.**
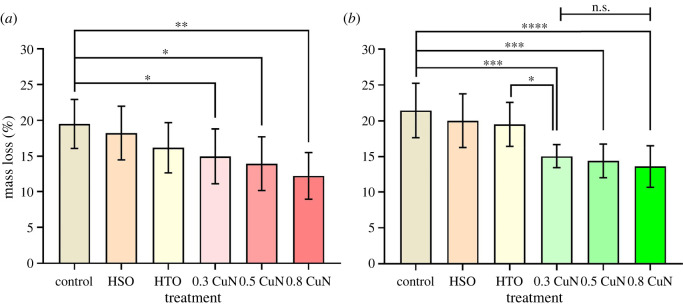
The average mass loss for bamboo exposed to *T. versicolor* (*a*) and *G. trabeum* (*b*). Asterisks represent statistical significance (one-way ANOVA followed by uncorrected Fisher's LSD multiple comparisons test, * *p* < 0.05; ** *p* < 0.01; *** *p* < 0.001; **** *p* < 0.0001) and n.s. indicates no significance. Error bars represent standard deviation of the mean.

When bamboo was heated in silicon oil (HSO in figure 7), the average ML dropped by 1.3% for the two test fungi. The slightly elevated decay resistance of HSO was attributed to both the heat treatment and the silicon oil attaching on bamboo surface [38], which retarded both water and decay fungi to some extent, but this effect was limited and only presented in short period. As the decay fungi adapted and found ways inside, the untreated inner part would be destroyed, leading to increased mass loss. Mineral oil, which contains no antifungal ingredients, when absorbed in bamboo under heat-cold treatment (HTO in figure 7), the average ML caused by *T. versicolor* decreased by 2% more than HSO, but similar to HSO in restraining *G. trabeum*. Thus, the resistance of mineral oil against decay fungi was selective and the effect was quite limited.

By contrast, bamboo heat-cold impregnated with CuN displayed improved resistance against *T. versicolor*, and this effect was enhanced with the increasing concentration of CuN (figure 7). Unfortunately, there were no statistically significant differences (*p* < 0.05) between HTO and three CuN treatments. However, significant differences were observed when control was compared with 0.3 CuN, 0.5 CuN and 0.8 CuN, which means that the combination of heat-cold method and CuN impregnation did improve the decay resistance of round bamboo against *T. versicolor*.

As for decay caused by *G. trabeum*, statistically significant differences were detected when control, HSO and HTO were compared with three CuN treatments, respectively. By contrast, no significant differences were observed when control was compared with HSO and HTO, respectively. Interestingly, there were no significant differences (n.s.) in multiple comparison of 0.3 CuN, 0.5 CuN and 0.8 CuN. This result suggested that the enhancement of decay resistance against *G. trabeum* mainly attribute to the incorporated CuN, instead of silicon and mineral oil. Besides, the increasing concentration of CuN showed insignificant contribution on the improvement of decay resistance against *G. trabeum*.

The treated blocks restrained white rot fungus *T. versicolor* and brown rot fungus *G. trabeum* efficiently, but the mass loss of which was beyond 12.2% even at the largest retention, 0.39 kg⋅m^−3^ of CuN. Hence, higher retention of copper might be required to achieve more desirable decay resistance. It has been reported that bamboo oriented strand board with phenol formaldehyde (PF) as the adhesive could reach a ML below 10% against *G. trabeum* and *T. versicolor* at a retention of CuN (calculated as copper) above 0.96 kg⋅m^−3^ [39]. When neem oil was added at a concentration of 25% into 0.3% CuN/kerosene oil, a desirable protection with a mass loss of 3.27% could be obtained against white rot fungus *Polyporus versicolor* [40]. It has been reported that a retention of 1.5 and 2.0 kg⋅m^−3^ CuN (caculated as copper) is sufficient to reduce the mass loss of red oak (*Quercus rubra*) below 10% when subjected to *T. versicolor* and *G. trabeum*, respectively [13].

Although bamboo is reported to be relatively more resistant against decay fungi than most of fast growing wood [41], higher than 0.39 kg⋅m^−3^ retention of CuN is required. For other copper-based preservatives such as ammoniacal copper quaternary (ACQ), chromated copper arsenate (CCA) and copper azole (CuAz), a retention above 1.17 kg⋅m^−3^ could reduce the mass loss of bamboo below 2.0% against both the white and brown rot fungi [5].

Additionally, it is worth noting that in the programmed heat-cold procedure of this experiment, no splitting or cracks were noticed, but in oven drying, the temperature should be lower than 40°C in the case of splitting [42,43]. Therefore, heat-cold treatment is currently an appropriate way to increase penetration and protection of round bamboo from decay.

### 3.5. Mould resistance

**Figure 8.**
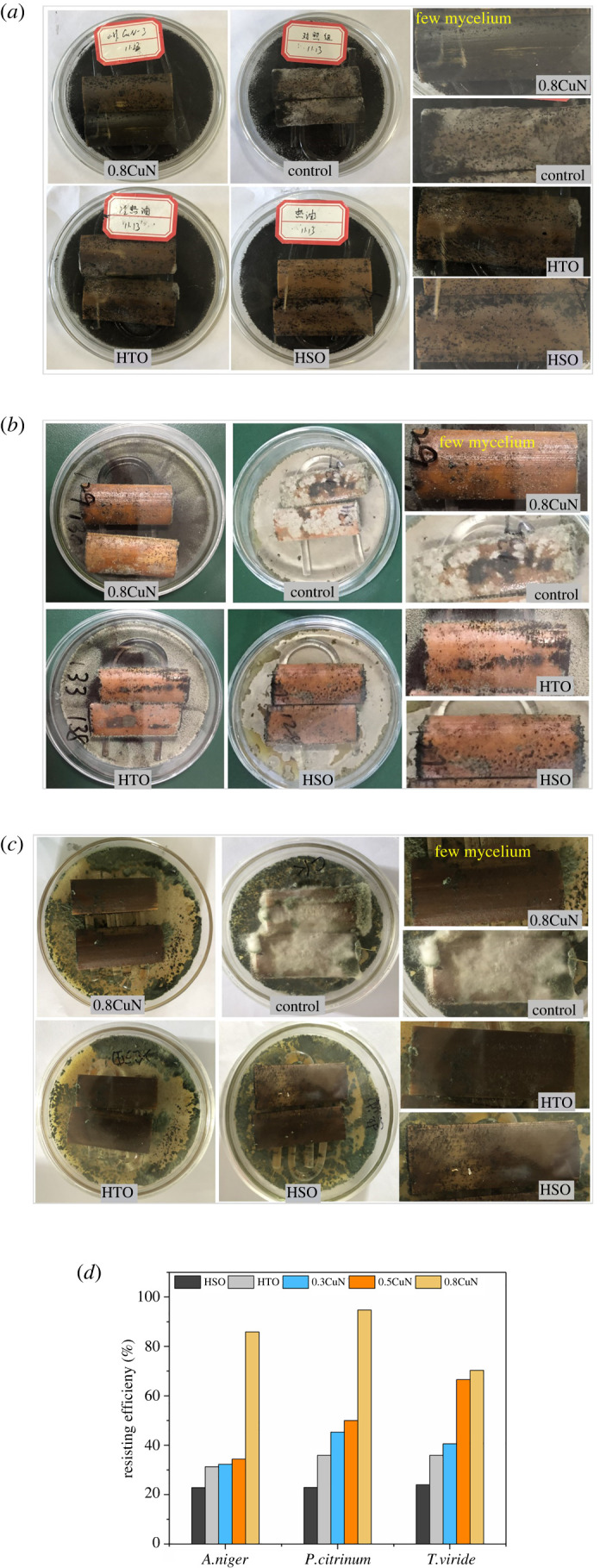
Images (*a–c*) and resisting efficacy (*d*) of untreated and treated bamboo against mould fungi after 30 days standard test [17]. (*a*) Images of bamboo stained by *A. niger.* (*b*) Images of bamboo stained by *P. citrinum.* (*c*) Images of bamboo stained by *T. viride.* (*d*) Resisting efficacy.

Bamboo is prone to contamination by mould fungi, which causes series problems and waste of bamboo resources. The resistance of bamboo against three mould fungi was examined and the results were exhibited in figure 8.

The untreated controls were completely covered with mould fungi within 10 days of cultivation. Both the hot oil-bathed bamboo (HSO in figure 8) and mineral oil impregnated bamboo (HTO in figure 8) could resist mould fungi to some extent, but lower than CuN treatments. Among the selected mould fungi, *A. niger* was the most aggressive one. Bamboo bathed in hot oil (HSO), heat-cold suction of mineral oil (HTO) or low concentration of CuN (0.3 CuN and 0.5 CuN) could not effectively resist *A. niger* unless a higher concentration of CuN was applied. HSO also presented as low as 24.0% and 22.9% resisting efficacy (RE) against *T. viride* and *P. citrinum*, indicating that the hydrophobic silicon oil alone could not protect bamboo from mould effectively. When absorbing mineral oil inside bamboo (HTO), the RE against *T. viride* and *P. citrinum* increased by 12.0%, displaying a promoted mould resisting effect after heat-cold absorption of mineral oil alone. When the mineral oil-based CuN was introduced in bamboo under heat-cold treatment, high RE was achieved.

The RE of bamboo was enhanced with an elevated concentration of CuN. Bamboo absorbing 0.8 CuN presented the best mould resistance, the RE soaring as high as 85.9% for *A. niger*, 94.8% for *P. citrinum* and 70.3% for *T. viride*, which was better than copper azole (CuAz) (concentration 1.0%) treated moso bamboo in resisting the three test fungi and comparable to ammoniacal copper quaternary (ACQ) (concentration 1.0%) in resisting *A. niger* and *P. citrinum* [44]. By contrast with copper-based chemicals, organic biocides present higher efficiency in resisting mould fungi, particularly, iodopropynyl butyl carbamate, dichlorooctyl isothiazolinone, propiconazole and tebuconazole [45]. Hence, for round bamboo in this case, the concentration of CuN should exceed 0.8% to inhibit the development of hypha as well as spore production, providing adequate protection to bamboo.

## 4. Conclusion

Heat-cold treatment was adopted in this study to impregnate CuN preservative into round bamboo, which was proved to be a feasible and effective method for round bamboo protection at present considering its typical structure and splitting characteristics.

The application of heat-cold treatment could improve the permeability of bamboo. CuN preservative was found to be distributed along the axial direction and deep in round bamboo. The fixation of CuN increased with the increment of retention, when the highest concentration of CuN 0.8 wt% was applied, the retention and fixation of Cu in round bamboo was 0.39 kg⋅m^−3^ and 85.3%, respectively. The SEM-EDX showed a slightly elevated amount of copper from the end inward, mainly in the cell cavity and intercellular spaces. Results of XPS and FTIR indicated that CuN was probably reacting with bamboo constitute, but no oxidation of bamboo was determined, which is probably due to the low heating temperature.

Both the decay and mould resistances were enhanced significantly for CuN-treated round bamboo in comparison with the untreated control, the heat oil treatment and the heat-cold impregnation of mineral oil alone. Therefore, heat-cold impregnation of CuN is expected to be one of the promising ways for manufacturing highly durable round bamboo. However, more research work should be conducted on the penetration of nodes as well as large size of round bamboo, for example 2 to 8 m or more in length, to verify the feasibility and effectiveness of this method.

## Data Availability

Data are available from the Dryad Digital Repository: https://doi.org/10.5061/dryad.r7sqv9sdw [46].

## References

[1] Lee CH, Yang TH, Cheng YW, Lee CJ. 2018 Effects of thermal modification on the surface and chemical properties of moso bamboo. Constr. Build. Mater. **178**, 59-71. (10.1016/j.conbuildmat.2018.05.099)

[2] Sun FL, Prosper NK, Wu HP, Qian JJ, Yang XS, Rao J, Guo M. 2017 A review on the development of wood and bamboo preservation. J. For. Eng. **2**, 1-8. (10.13360/j.issn.2096-1359.2017.05.001)

[3] Rao J, Jiang J, Prosper NK, Yang XS, Liu TS, Cai W, Wang H, Sun FL. 2019 Combination of polyethylene glycol impregnation and paraffin heat treatment to protect round bamboo from cracking. R. Soc. Open. Sci. **6**, 190105. (10.1098/rsos.190105) 31827815 PMC6894564

[4] Yang TH, Lee CH, Lee CJ, Cheng YW. 2016 Effects of different thermal modification media on physical and mechanical properties of moso bamboo. Constr. Build. Mater. **119**, 251-259. (10.1016/j.conbuildmat.2016.04.156)

[5] Wang YM, Liu JL, Jiang ML, Qin DC. 2007 Decay resistance of bamboo treated with different preservatives. Wood. Ind. **21**, 8-10.

[6] Salamah S, Dahlan JM. 2008 Vacuum-pressure treatment of rubberwood (*Hevea brasiliensis*) using boron-based preservative. J. Trop. For. Sci. **20**, 1-7.

[7] Rabbi MF, Islam MM, Rohman ANMM. 2015 Wood preservation: improvement of mechanical properties by vacuum pressure process. Inter. J. Eng. Appl. Sci. **2**, 2394-3661.

[8] Tripathi S, Poonia PK. 2015 Treatability of Melia composita using vacuum pressure impregnation. Maderas. Cienc. tecnol. **17**, 373-384. (10.4067/S0718-221X2015005000035)

[9] Goodell B, Nicholas DD, Schultz TP. 2003 Wood deterioration and preservation: advances in our changing world. In Wood deterioration and preservation, pp. 2-6. Washington, DC: American Chemical Society.

[10] Fei BH, Tang T. 2019 Synergistic effects of tung oil and heat treatment on physicochemical properties of bamboo materials. World. Bamboo. Rattan. **17**, 73-77. 10.1038/s41598-019-49240-8 PMC6731322 31492890

[11] Liese W, Tang TKH. 2015 Preservation and drying of bamboo. Bamboo. **10**, 257-297. (10.1007/978-3-319-14133-6_9)

[12] Côté WA. 1968 Wood preservation. Princ. Wood Sci. Technol. **5**, 136-159. (10.1007/978-3-642-87928-9_5)

[13] Kamden DP, Fair R, Freeman M. 1996 Efficacy of water-borne emulsion of copper naphthenate as preservative for northern red oak (*Quercus rubra*) and soft maple (*Acer rubrum*). Holz. Roh-und. Werkst. **54**, 183-187. (10.1007/s001070050163)

[14] Kamdem DP, Pascal N, Herman D, Shu ZJ. 2020 Biological performance of a formulation containing water-dispersible copper naphthenate and sodium fluoride against decay fungi. Wood Mater. Sci. Eng. **15**, 30-36. (10.1080/17480272.2018.1463290)

[15] AWPA E11–2016. 2016 Standard method for accelerated evaluation of preservative leaching. Birmingham, UK: AWPA Book of Standards. American Wood Protection Association.

[16] AWPA E10-2016. 2016 Laboratory method for evaluating the decay resistance of wood-based materials against pure basidiomycete cultures: soil/block test. Birmingham, AL: AWPA Book of Standards. American Wood Protection Association.

[17] AWPA E24-2021. 2021 Laboratory method for evaluating the mold resistance of wood-based materials: mold chamber test. Birmingham, AL: AWPA Book of Standards. American Wood Protection Association.

[18] AWPA M4-2015. 2015 Standard for the care of preservative-treated wood products. Birmingham, AL: AWPA Book of Standards. American Wood Protection Association.

[19] Bao MZ, Yu WJ, Chen YH, Yu YL, Wu ZX, He S, Li N. 2020 Effects of CuAz preservative on the antiseptic performance and physical and mechanical properties of poplar scrimber. J. Zhejiang. A&F. Uni. **37**, 165-170. (10.11833/j.issn.2095-0756.2020.01.022)

[20] Wang YM, Liu JL, Wang XM. 2008 Decay and leaching resistance of bamboo treated with ACQ preservatives. China. Wood. Ind. **22**, 14-16.

[21] Chen GW, Luo HY. 2020 Effects of node with discontinous hierarchical fibers on the tensile fracture behaviors of natural bamboo. Sustain. Mater. Techno. **26**, e00228. (10.1016/j.susmat.2020.e00228)

[22] Lv HF, Chen XF, Liu XM, Fang CH, Liu HR, Zhang B, Fei BH. 2018 The vacuum-assisted microwave drying of round bamboos: drying kinetics, color and mechanical property. Mater. Lett. **223**, 38. (10.1016/j.matlet.2018.04.038)

[23] Sun FL, Duan XF, Mao SF, Wen GF, Wang SS. 2007 Decay resistance of bamboo wood treated with chitosan-metal complexes against the white-rot fungus *Coriolous versicolor*. Sci. Silvae. Sin. **43**, 83-87.

[24] Baileys EK, Baileys RT. 1993 A report on southern pine utility poles treated with copper naphthenate. *Proc. American Wood-Preservers' Assoc.* **88**, 268–288.

[25] Hein RW. 1987 Mobility of copper naphthenate in soil. Cleveland, OH: Mooney Chemicals Inc. Laboratory memorandum.

[26] Hein RW. 1990 Mobility of copper napthenate in soil. Cleveland, OH: Mooney Chemicals Inc. Laboratory memorandum. 3601-M.

[27] Lebow S. 1996 Leaching of wood preservative components and their mobility in the environment – summary of pertinent literature. Gen. Tech. Rep. FPL-GTR-93. Madison, WI: U.S. Department of Agriculture, Forest Service, Forest Products Laboratory.

[28] Luo JJ. 2020 Comparative anatomy of bamboo metaxylem vessel pits. Chin. Acad. For. **2**, 29–31. (10.27625/d.cnki.gzlky.2020.000206)

[29] Tang T, Chen XF, Zhang B, Liu XM, Fei BH. 2019 Research on the physico-mechanical properties of moso bamboo with thermal treatment in tung oil and its influencing factors. Materials. **12**, 599. (10.3390/ma12040599) 30781544 PMC6416738

[30] Rubina MS, Vasil'kov AY, Naumkin AV, Shtykova EV, Abramchuk SS, Alghuthaymi MA, Abd-Elsalam KA. 2017 Synthesis and characterization of chitosan-copper nanocomposites and their fungicidal activity against two sclerotia-forming plant pathogenic fungi. J. Nanostructure. Chem. **7**, 249-258. (10.1007/s40097-017-0235-4)

[31] Matuana LM, Kamdem DP. 2002 Accelerated ultraviolet weathering of PVC/wood-flour composites. Polym. Eng. Sci. **42**, 1657-1666. (10.1002/pen.11060)

[32] Okon KE, Udoakpan UI. 2019 Physicochemical properties of *Pinus massoniana* wood subjected to silicone oil heat treatment. Maderas. Cienc. Tecnol. **21**, 531-544. (10.4067/s0718-221x2019005000409)

[33] Cheng DL, Li T, Smith GD, Xu B, Li YJ. 2018 The properties of *Moso* bamboo heat-treated with silicon oil. Eur. J. Wood. Wood. Prod. **76**, 1273-1278. (10.1007/s00107-018-1301-4)

[34] Rana R, Langenfeld-Heyser R, Finkeldey R, Polle A. 2010 FTIR spectroscopy, chemical and histochemical characterisation of wood and lignin of five tropical timber wood species of the family of Dipterocarpaceae. Wood. Sci. Technol. **44**, 225-242. (10.1007/s00226-009-0281-2)

[35] Meng FD, Yu YL, Zhang YM, Yu WJ, Gao JM. 2016 Surface chemical composition analysis of heat-treated bamboo. Appl. Surf. Sci. **371**, 383-390. (10.1016/j.apsusc.2016.03.015)

[36] Kumar A, Ryparovà PP, Kasal B, Adamopoulo S, Hajek P. 2018 Resistance of bamboo scrimber against white-rot and brown-rot fungi. Wood. Mater. Sci. Eng. **15**, 57-63. (10.1080/17480272.2018.1475420)

[37] Eriksson KE. 1978 Enzyme mechanisms involved in cellulose hydrolysis by the rot fungus *Sporotrichum pulverulentum*. Biotechnol. Bioeng. **20**, 317-332. (10.4067/s0718-221x2019005000409)

[38] Lyon F, Thevenon MF, Hwang WJ, Imamura YJ, Gril J, Pizzi A. 2007 Effect of an oil heat treatment on the teachability and biological resistance of boric acid impregnated wood. Ann. Forest. Sci. **64**, 673-678. (10.1051/forest:2007046)

[39] Wei WS, Qin DC, Jin JW, Ding X, Gan J. 2011 Decay resistance of bamboo oriented strand boards treated with preservative. Wood. Ind. **25**, 8-21.

[40] JitKaur P, Satya S, Pant KK, Naik SN. 2015 Eco-friendly preservative treated bamboo culm: compressive strength analysis. Int. Sch. Sci. Res. Innov. **9**, 43-46. (10.15242/ijrcmce)

[41] Wei DS, Schmidt O, Liese W. 2013 Method to test fungal degradation of bamboo and wood using vermiculite as reservoir for moisture and nutrients. Maderas. Cienc. Tecnol. **15**, 349-356. (10.4067/S0718-221X2013005000027)

[42] Vetter RE, Sá Ribeiro RA, Sá Ribeiro MG, Miranda IPA. 2015 Studies on drying of imperial bamboo. Eur. J. Wood. Prod. **73**, 411-414. (10.1007/s00107-015-0900-6)

[43] Yan W, Zhang B, Fu WS, Zhou JB. 2017 Strain characterization and mechanism study on annular shrinkage of bamboo culm (*Phyllostachys pubescens*). China Forest. Prod. Ind. **44**, 16-26.

[44] Zhang J, Yuan SF, Fan H, Li Q, Wang HY. 2016 Effect of different antimildew and antiseptic agents on reconstructed bamboo timber. J. Zhejiang. For. Sci. Tech. **36**, 8-12.

[45] Zhang LS, Qin DC, Ren HL. 2013 Study on mold control effectiveness of bamboo treated with organic fungicides. Forest. Mach. Woodworking. Equip. **41**, 23-25.

[46] Jiang J, Han SB, Ren X, Wang H, Yu HW, Sun FL. 2022 Data from: Enhanced durability of round bamboo treated with copper naphthenate under heat-cold impregnation. *Dryad Digital Repository.* (10.5061/dryad.r7sqv9sdw) PMC9682299 36425518

